# Comparative evaluation of diode laser over standard procedure managing endo-perio lesions

**DOI:** 10.6026/973206300212825

**Published:** 2025-08-31

**Authors:** Jitender Sharma, Manoj Kumar Meena, Rakashree Chakraborty, Shruthi M.S., Shalini H.S., Richa Goel, Siddhartha Das, Munaz Mulla

**Affiliations:** 1 Department of Periodontics & Oral Implantology, Vyas Dental College & Hospital, Jodhpur, Rajasthan, India; 2 Department of Prosthodontics, Daswani Dental College and Research Centre, Kota, Rajasthan, India; 3 Department of Oral Medicine and Radiology, Maharishi Markandeswar College of Dental Sciences and Research, Mullana, Ambala, Haryana, India; 4 Department of Periodontics, Subbaiah Institute of Dental Sciences, Shivamogga, Karnataka, India; 5 Department of Public Health Dentistry, Karnavati School of Dentistry, Karnavati University, Gandhinagar, Gujarat, India; 6 Intern, Kalinga Institute of Dental Science, Kalinga Institute of Industrial Technology (KIIT) Deemed to be University, Patia, Bhubaneswar, Odisha, India; 7 Department of Periodontics, Saveetha Dental College and Hospitals, Saveetha Institute of Medical and Technical Sciences, Saveetha University, Chennai, India

**Keywords:** Diode laser, endo-perio lesions, periodontal therapy, root canal treatment

## Abstract

Endo-perio lesions are complicated clinical situations that present difficult clinical problems. Therefore, it is of interest to compare the efficacy of a standard treatment protocol over diode laser for management of endo-perio lesions. A total of 40 patients with endo-perio lesions were randomly assigned to two groups; standard treatment protocol, including root canal therapy with scaling and root planing (SRP) and augmented with diode laser therapy. At baseline and six months after treatment, clinical data such as radiographic bone density using digital radiography, clinical attachment level (CAL), bleeding on probing (BOP), and probing depth (PPD) were collected. Bacterial decrease was evaluated by microbiological analysis. Thus, clinical results are considerably improved when diode lasers are used as an adjuvant to established treatment protocols for endo-perio lesions.

## Background:

An inflammatory condition linked to bacterial plaque, periodontitis increases tooth morbidity by causing the loss of tooth-supporting tissue. The goal of treating periodontitis is to remove bacterial deposits from the tooth surface by controlling plaque, scaling and root planing (SRP). To lower the bacterial load in some circumstances, antimicrobial therapy must be used in conjunction with traditional treatment [1]. Endo-perio lesions are complicated clinical situations that present difficult clinical problems and are defined by the coexistence of endodontic and periodontal diseases in the same tooth. These lesions have a complex aetiology that frequently includes systemic diseases, trauma and microbial infection [2]. Combining endodontic therapy with periodontal care is the conventional approach to treating endo-perio lesions. Recent developments indicate that by lowering the microbial burden and encouraging tissue repair, the supplementary use of diode lasers may improve therapy results. Diode lasers, especially those with a wavelength of 940 nm, have drawn interest among these because of its antibacterial qualities, capacity to lower inflammation, and ability to aid in tissue healing [2, 3]. To convert electrical energy into light energy, a semiconductor laser called a diode laser (DL) is used with gallium, arsenide, aluminium and indium. It is safe to do surgery using a diode laser [1, 4]. When it comes to controlling soft tissues, the diode laser outperforms compared to other types of lasers. The diode laser has been utilised extensively in dentistry because of its affordability, biocompatibility and capacity to reduce the number of microorganisms [5]. Anaerobic microbes that live in periodontal pockets may usually be reduced by lasers [1]. Toxins and microorganisms can be effectively removed with diode lasers. The depth of periodontal pockets inhabited by anaerobic bacteria, which cause bone loss, is decreased by lasers [6]. The studies regarding clinical and microbiological efficacy of diode laser on endo-perio lesions is very scares. Therefore, it is of interest to evaluate the clinical, radiographic and microbiological impact of diode laser in periodontal treatment, compared to standard therapy.

## Materials and Methods:

Following ethical clearance and participant informed permission, a randomised controlled prospective clinical trial was carried out in the Periodontics and Endodontics department. 40 patients with endo-perio lesions were divided into two groups at random, each containing 20 samples: Group I (control) received standard treatment, which included root canal therapy and scaling and root planing (SRP); Group II (test) received SRP in addition to diode laser therapy (940 nm). The study comprised patients with endo-perio lesions who were not receiving systemic or periodontal therapy, were between the ages of 25 and 55, and had at least two afflicted sextants with a minimum pocket depth of 4-6 mm. Smokers, those with medical conditions, alcoholics, and those wearing orthodontic appliances were among the exclusion criteria. At baseline, both endodontic and periodontal findings were recorded. Following root canal therapy, all patients underwent a periodontal procedure using a rotary file and standard obturation technique. In group II, root canal disinfection was accomplished using a laser. Only root planning and scaling were carried out in the control group. Soft tissue diode lasers (Biolase Epicx Soft Tissue Dental Laser, BIOLASE, Inc., California, USA) were used in the test group's deepest pockets after periodontal probing and non-surgical treatment (SRP). A wavelength of 940 Nm, a frequency of 20 Hz, and one watt of power were used to irradiate the diode laser. Within the periodontal pockets, a single laser session was run for 15 seconds in a continuous up-and-down motion. For six months, the pockets were disinfected twice a month. At baseline and six months after treatment, clinical measures such as bleeding on probing (BOP), bone loss (BL), probing depth (PPD), and clinical attachment level (CAL) were measured. Digital radiographs that were subjected to imaging software analysis were used to measure changes in radiographic bone density. Using endodontic paper points that inserted up to the base of the chosen pockets in both groups, plaque samples were taken both before and after treatment follow-up visits. To guarantee proper plaque control, patients were advised to brush twice a day. Changes in bacterial count were evaluated by microbiological analysis. Gram-negative enteric bacteria are isolated by quantitatively analysing anaerobic bacteria using the MacConkey agar test. Gram-negative enteric bacteria are isolated using the MacConkey agar test, which also does quantitative analysis of anaerobic bacteria. The obtained data was statistically analysed using SPSS statistical software version 23.0 using IBM Corp., Armonk, NY, USA and Chi-square test, Mann-Whitney U-test at P<0.05.

## Results:

Table 1 (see PDF) indicated the clinical parameter at baseline to after treatment after 6 months of follow up. There was improvement in clinical and radiographic parameters in test group compared to control group which is statistically significant (p<0.001). Table 2 (see PDF) indicates reduction in periodontal pathogen after laser treatment after 6 months of follow up.

## Discussion:

Chronic periodontitis causes inflammation of the tooth-supporting structure that is exudative, proliferative, and degenerative. Dental plaque is the causative agent of periodontal disorders. Loss of bone and connective tissue is a hallmark of periodontitis [7]. Alveolar bone and the floor of the maxillary sinuses are among the tissues that are severely destroyed by marginal and periapical periodontal disorders. Increased periodontal pocket depth and connective tissue loss are symptoms of advanced periodontitis. The tooth's pulp is where secondary alterations start. The removal of pathogens from diseased root canals and periodontal pockets is a necessary part of treating endo-periodontal diseases [6]. The best way to eradicate the disease's root cause and lower the parameters of periodontitis is through conventional non-surgical therapy [1]. The current study results indicate that non -surgical periodontal treatment alone or in conjugation with a diode laser (940 nm) provides a significant improvement in clinical periodontal parameters [8]. We cannot determine the aetiology of the periodontitis, the patient's susceptibility to the disease, if the disease is worsening, or whether the therapy is working thanks to the clinical evaluation and X-ray analysis. Therefore, microbiological, immunological and genetic testing should also be taken into consideration when assessing the patient's periodontal state [9]. The findings of earlier studies that demonstrated that diode lasers stimulate osteoblast activity and enhance bone regeneration are further supported by radiographic bone density improvements in the laser-assisted group [10, 11]. Diode lasers' antibacterial effectiveness against important pathogens linked to endo-perio lesions has been shown in the current investigation. This is consistent with Behera *et al.*'s earlier research [2]. Moghadam came to the conclusion that both the microbial load and the post-endodontic discomfort were considerably reduced by root canal disinfection using depotphoresis and diode laser [12]. According to Dubey *et al.* postendodontic discomfort was considerably reduced by diode laser root canal disinfection [13]. Faragalla *et al.* assessed the diode laser's clinical and microbiological effects in the treatment of instances of periodontitis. They came to the conclusion that the clinical parameters could be improved with diode laser treatment [1]. Diode laser treatment was more successful in lowering periodontal clinical indicators, according to Sopi *et al.* [7]. Behera *et al.* assessed how effective standard methods were in comparison to those enhanced with a diode laser. They came to the conclusion that treating endo-perio lesions with a 940 nm diode laser as an adjuvant greatly improves clinical results [2]. Using a diode laser, Dembowska *et al.* discovered a decrease in pocket depth [6]. Our findings are consistent with the results. Lai *et al.* found no variations in the assessed clinical parameters or radiographic findings between the test (He-Ne laser) and control sites at 3 to 12 months, which is in contrast to the current study's findings [14]. Effective management of endoperio lesions is aided by proper endodontic treatment, following by periodontal therapy with SRP and diode laser therapy. Furthermore, although the 940 nm diode laser was the main focus of this investigation, comparative research using different laser types or wavelengths may offer further information about how to best optimise treatment regimens.

## Conclusion:

A diode laser (940 nm) can significantly reduce inflammation, the depth of periodontal pockets, and the presence of microorganisms when used in conjunction with SRP.

## References

[1] Faragalla AI (2021). J Res Med Dent Sci..

[2] Behera S (2024). Frontiers in Health Informatics..

[3] Cobb CM (2006). J Periodontol..

[4] Adriaens PA (2004). Periodontology..

[5] Borrajo JL (2004). Photomed Laser Surg..

[6] Dembowska E (2022). J. Clin. Med..

[7] Sopi M (2020). The Open Dentistry Journal..

[8] Seyed-Monir A (2023). Dent Res J (Isfahan)..

[9] Ebersole JL (2015). Front Cel Infec Microbio..

[10] Aoki A (2004). Periodontol 2000..

[11] Chan Y, Lai CH (2003). Lasers Med Sci..

[12] Moghadam MD (2021). Giornale Italiano di Endodonzia..

[13] Dubey P (2024). J Pharm Bioall Sci..

[14] Lai SM (2009). Photomed Laser Surg..

